# Image Registration-Based Bolt Loosening Detection of Steel Joints

**DOI:** 10.3390/s18041000

**Published:** 2018-03-28

**Authors:** Xiangxiong Kong, Jian Li

**Affiliations:** Department of Civil, Environmental, and Architectural Engineering, University of Kansas, Lawrence, KS 66045, USA; jianli@ku.edu

**Keywords:** bolt loosening detection, intensity-based image registration, feature matching, structural health monitoring, structural inspection, superpixel, civil structures, steel joints, feature tracking

## Abstract

Self-loosening of bolts caused by repetitive loads and vibrations is one of the common defects that can weaken the structural integrity of bolted steel joints in civil structures. Many existing approaches for detecting loosening bolts are based on physical sensors and, hence, require extensive sensor deployment, which limit their abilities to cost-effectively detect loosened bolts in a large number of steel joints. Recently, computer vision-based structural health monitoring (SHM) technologies have demonstrated great potential for damage detection due to the benefits of being low cost, easy to deploy, and contactless. In this study, we propose a vision-based non-contact bolt loosening detection method that uses a consumer-grade digital camera. Two images of the monitored steel joint are first collected during different inspection periods and then aligned through two image registration processes. If the bolt experiences rotation between inspections, it will introduce differential features in the registration errors, serving as a good indicator for bolt loosening detection. The performance and robustness of this approach have been validated through a series of experimental investigations using three laboratory setups including a gusset plate on a cross frame, a column flange, and a girder web. The bolt loosening detection results are presented for easy interpretation such that informed decisions can be made about the detected loosened bolts.

## 1. Introduction

Bolted steel joints are among the most common types of steel connections and have been widely applied in various civil structures such as buildings and bridges. Bolted steel joints are prone to structural damage over long service periods due to self-loosening of the bolts, which is mainly caused by repetitive loads and/or vibrations. Bolt loosening leads to a loss of clamping force acting on the joint, further causing stiffness degradation and potentially structural failure [1]. Therefore, monitoring the health condition of bolts in a timely fashion is essential for structural integrity as appropriate replacements or retrofits can then be applied before the steel joints reach critical conditions.

Human visual inspection has been commonly applied for detecting bolt loosening in civil structures. For example, the Federal Highway Administration (FHWA) [2] requires routine inspections with two-year intervals for highway bridges in the United States. Trained bridge inspectors visually detect and record various structural defects in bridges, including bolt loosening, during inspections. However, human inspection is labor intensive and less effective as bolts may become loose between the inspections. More importantly, inspection results may contain errors due to inconsistencies in inspection skills and abilities to interpret data among inspectors. For example, Graybeal et al. [3] performed an experimental study to evaluate the inspection skills of bridge inspectors using bridges in South Central Pennsylvania and Northern Virginia in the United States. For a particular bolt loosening defect, only 19 out of 42 inspectors successfully identified the deficiency.

Advanced bolt loosening detection technologies have been developed in the fields of both structural health monitoring (SHM) and nondestructive testing (NDT). Yang and Chang [4] proposed an attenuation-based diagnostic method through an ultrasonic technique to detect bolt loosening in space operation vehicles. Zhao et al. [5] adopted a piezoelectric-based sensor network for identifying damage of a riveted panel in an aircraft wing. A similar piezoelectric-based approach was applied by Okugawa [6] through a smart washer. In addition, Wu et al. [7] developed a radio-frequency identification (RFID)-based sensing method to detect bolt loosening in coal mine structures. Nevertheless, the success of these methods relies on extensive work of human operations and/or sensor deployments, which could be costly and less flexible for rapid inspections of bolted steel joints in civil structures. Reference [8] offers a comprehensive literature review of various technologies for bolt loosening detection.

Computer vision-based technologies have received significant attention in the SHM community due to the benefits of being low cost, easy to deploy, and contactless. Several vision-based approaches have been reported for monitoring health conditions of civil structures on both global and local scales. Some of the recent applications of vision-based SHM include structural system identification [9,10,11], displacement monitoring [12,13,14], post-disaster structural classification [15], damage detection [16], and fatigue crack identification [17,18]. Furthermore, when equipped with autonomous platforms such as unmanned aerial vehicles (UAVs), vision-based SHM could bring higher flexibility and cost effectiveness to structural inspections. Recently, UAVs have been applied in structural system identification [19], structural inspection of buildings [20], water treatment plants [21], bridges [22], and so forth. A state-of-the-art review of vision-based SHM in civil structures can be found in [23].

Despite the recent successes of vision-based SHM, limited work is found in the context of vision-based bolt loosening detection. The early related work reported in the literature was performed by Park et al. [24] and Park et al. [25]. In these two studies, a Hough transform-based edge detection technique was adopted to extract the boundaries of the bolt nuts in multiple images. The loosened bolt can be detected by identifying the rotation of boundaries. However, the described approaches might rely on extensive operations for comparing the nut boundaries (i.e., edges) before and after the nut rotation, which would limit its flexibility for automatically processing a large volume of images. Cha et al. [26,27,28] integrated machine learning with vision-based bolt dimension extraction, introducing a robust method for detecting loosened bolts. The study in [26] reported a detection accuracy of 87.5% based on a small set of training images, including four loosened and four tightened bolts. Nevertheless, prior knowledge about the damage state of the bolt (i.e., classifications of loosened bolts and tightened bolts) are needed to train the classifier and the training procedure would have to be repeated in the case of new types of bolts with different dimensions or shapes.

This paper presents a new vision-based bolt loosening detection method that uses image registrations. Instead of finding the rotation of the nut’s boundaries [24] or building classifiers based on the dimensions of the bolt head [26], directly mapping images at different inspection periods into the same coordinate system and uncovering differential features caused by the loosened bolt could form a more straightforward solution. Compared with previous vision-based bolt loosening detection methods, our approach does not require extensive operations for finding the rotation of the nut’s boundaries and does not require prior knowledge about the monitored structure (such as bolt types) or damage states of the bolt. In these regards, our approach would be more flexible and cost effective for engineering applications. The detection results of this approach are also presented for easy interpretation such that direct actionable decisions can be made to conduct condition-based maintenance procedures.

The rest of this paper is organized as follows: Section 2 demonstrates the proposed methodology along with its technical details; Section 3 validates the approach through three experimental tests; Section 4 further investigates the robustness of the proposed approach; Section 5 discusses the computation cost and limitations; Section 6 concludes this study.

## 2. Methodology

**Figure 1:**
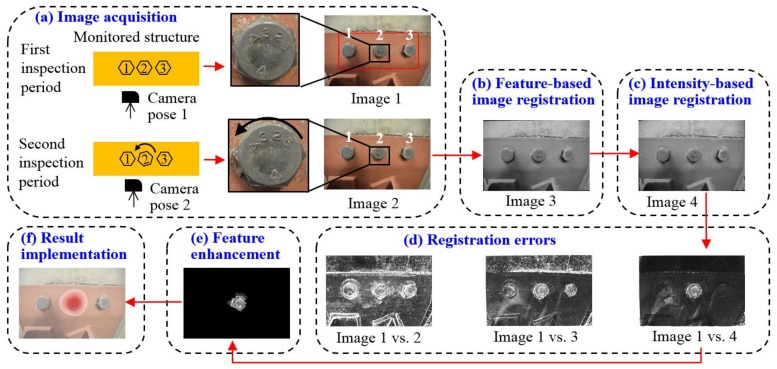
Methodology of the proposed bolt loosening detection: (**a**) image acquisition; (**b**) feature-based image registration; (**c**) intensity-based image registration; (**d**) registration errors; (**e**) feature enhancement; and (**f**) result implementation. The brightness of images Figure 1d,e is enhanced for demonstration purposes. The red solid arrows indicate the flow of this methodology.

Figure 1 demonstrates the overall methodology of our approach with detailed discussions provided in the rest of this section. As an illustration, suppose a bolted steel joint, termed as the monitored structure in Figure 1a, is evaluated at two inspection periods and Bolt 2 is loosened during the inspection interval. Two input images, denoted as Image 1 and 2, are collected by a digital camera at the two inspection periods. Since the camera poses of the two images would not necessarily be identical, directly identifying the rotated bolt by overlapping the two input images would be challenging. This is confirmed by the intensity comparison between Image 1 and 2, as shown in Figure 1d, in which intensities of exactly matched pixels are illustrated as 0 (black) and intensities of unmatched pixels are in the region of 1 to 255 (grey to white), deepening the level of their discrepancies.

In order to align two input images, we first adopt a feature-based image registration method (Figure 1b) to transform Image 2 into a new image, denoted as Image 3, so that Image 3 and 1 could share the same coordinate system. A region of interest (ROI) should be assigned prior to this procedure as shown in the red block in Image 1. The purpose of defining the ROI is to specify a region in Image 1 as the target region where Image 2 should match. Generally, the ROI should only cover a group of bolts and their adjacent structural surface and exclude unnecessary elements in the scene (e.g., the wall in the background in Image 1). After the feature-based image registration, the matching performance is improved as shown in the intensity comparison between Image 1 and 3 (Figure 1d). Nevertheless, misalignments (i.e., registration errors) still exist, especially around the areas of Bolts 1 and 3, even though they are intact during the inspection interval. Such errors commonly exist in feature-based image registration and a detailed discussion about these errors can be found in [29].

To reduce registration errors further, an intensity-based image registration method is applied to Image 3 (Figure 1c), allowing Image 3 to be non-rigidly matched to Image 1. The newly transformed image is now denoted as Image 4. The intensity comparison between Image 1 and 4 is seen in Figure 1d. Registration errors could be significantly reduced around Bolts 1 and 3, while still exist in the loosened bolt (Bolt 2) due to the bolt rotation. We treat these errors as the bolt loosening features that are introduced by multiple sources during the bolt rotation, such as hexagon boundaries of the bolt head, the mark of A325, and other surface textures on the bolt head surface. Next, we further enhance the above bolt loosening features by filtering out adjacent noisy content (Figure 1e) in the registration errors. Finally, we map the enhanced bolt loosening features to the original input image (Image 1) so that the loosened bolt can be directly visualized (Figure 1f). This would allow informed actionable decisions to be made regarding performing appropriate rehabilitations and/or retrofitting to the monitored steel joint.

Two image registration processes serve as the key components in the proposed approach. The feature-based image registration effectively aligns two input images into the same coordinate system based on a predefined ROI. However, small misalignments are usually associated with feature-based image registration. The intensity-based image registration, on the other hand, is able to adjust small misalignments but may have difficulties handling significant misalignments if the input images are taken from very different camera poses. By adopting these two image registration processes in a successive manner, the misalignments between two input images can be gradually reduced through each registration process. It should be noted that the algorithm for feature-based image registration is not tied to a particular intensity-based image registration method and vice versa. Section 4.3 demonstrates several other approaches to perform feature-based image registration. For intensity-based image registration, besides the method adopted in this study, other well-established approaches can also be found in [30].

### 2.1. Image Acquisition

A consumer-grade digital camera is required for image acquisition. In this study, we adopted a Nikon D7100 camera and a Sigma 17–50 mm lens with the auto-shooting mode unless stated otherwise. The distance between the camera and the monitored structure relies on the resolution of the camera; a typical distance of 20 to 50 cm was adopted in this study. The camera can be held by hands during image acquisition and the images should directly capture the detected bolt and its adjacent structural surface without any obstructions. Ambient lighting conditions are generally acceptable. The image plane can be either parallel or skew to the monitored structural surface (see Test 3 in Section 3 for a detailed discussion on the skew case). When collecting the images at different inspection periods, the lighting conditions and camera pose should be similar between inspection periods in order to produce the optimal result. Camera calibration is not required in this study.

### 2.2. Feature-Based Image Registration

The purpose of feature-based image registration is to align two images into the same coordinate system using matched features (i.e., correspondences). For this approach to be viable, features (also known as feature points, corner points, or key points) are first detected in both input images. Then, a matching algorithm is adopted to find matched features between the two images, based on which a geometric transformation matrix can be estimated to transform the second image to the coordinate system of the first image.

**Figure 2:**
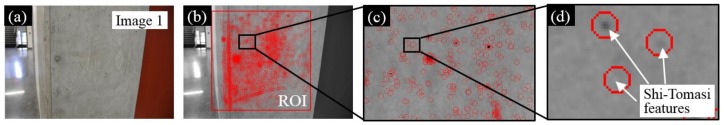
Demonstration of feature detection using an image of a concrete column. (**a**) First input image; (**b**) 15,117 feature points are detected in the ROI; (**c**) detailed look of Figure 2b; and (**d**) detailed look of Figure 2c. Red circles in Figure 2b–d are Shi–Tomasi features.

To better demonstrate this procedure, an example is illustrated using two input images of a concrete column taken by the aforementioned digital camera with a resolution of 6000 pixels × 4000 pixels. Suppose we wanted to match the front face of a column in two input images. First, denoted the first input image as Image 1 (Figure 2a) and an ROI (3500 pixels × 3500 pixels) in Image 1 is selected to cover the front face of the column. Next, the Shi–Tomasi algorithm [31] is adopted to extract features and the detected features are denoted as Feature set 1. This feature extraction procedure is flexible and can be achieved by many other feature types as well. A detailed comparison of the different features for feature-based image registration can be found in Section 4.3. As can be seen in Figure 2d, Shi–Tomasi features (highlighted by red circles) are based on the unique intensity change at a localized region in both the horizontal and vertical directions, which is the intrinsic nature existing in most images.

**Figure 3:**
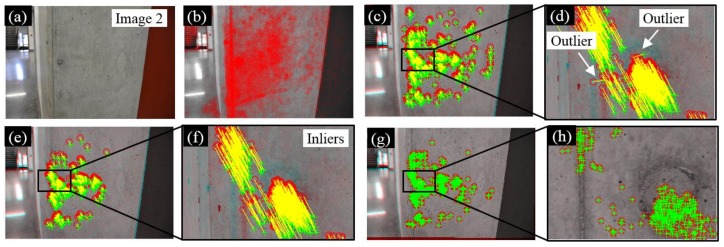
Demonstration of feature matching. (**a**) Second input image of the concrete column; (**b**) Shi–Tomasi features in Image 2; (**c**) matched feature points between Image 1 and 2; (**d**) a detailed look of Figure 3c; (**e**) inliers for estimating the transformation matrix; (**f**) a detailed look of Figure 3e; (**g**) inliers after image mapping; and (**h**) a detailed look of Figure 3g. Red circles in Figure 3c–h are features in Image 1, while green crosses are features in Image 2.

Figure 3a illustrates the second input image of the concrete column using a different camera pose, denoted as Image 2. Similarly, Shi–Tomasi features are extracted for the entire region of Image 2, denoted as Feature set 2 in Figure 3b. Next, we adopt the Kanade–Lucas–Tomasi (KLT) tracker [32,33] to match each point in Feature set 1 to any potential point in Feature set 2. As a result, 1370 matched features can be found in Figure 3c where red circles are features in Image 1 and green crosses represent features in Image 2. Among all the matched features, some outliers can be found (Figure 3d), indicating matching failures. These outliers can be further eliminated utilizing the maximum likelihood estimation sample consensus (MLESAC) algorithm proposed by Torr and Zisserman [34] and the new matched results (i.e., inliers) are shown in Figure 3e,f. In total, 1175 matched features can be found, based on which a projective geometric transformation matrix can be estimated so that Image 2 can be registered to the coordinate system of Image 1. The projective geometric transformation can remove the projective distortion between Image 1 and 2 taken under different camera poses. Matched feature points after image registration can be found in Figure 3g,h where the red circles match the green crosses.

### 2.3. Intensity-Based Image Registration

**Figure 4:**
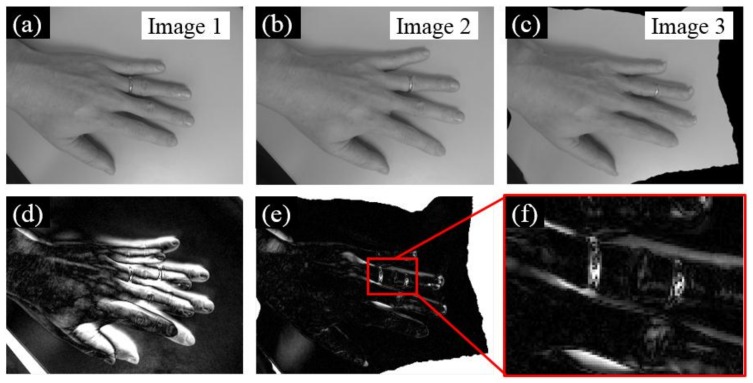
Demonstration of intensity-based image registration through an example of two images of the same hand under different poses. (**a**,**b**) Two input images; after registration, Image 2 is registered as Image 3 as shown in (**c**); (**d**) the intensity comparison between Image 1 and 2; (**e**) the intensity comparison between Image 1 and 3; and (**f**) a detailed look of Figure 4e.

The purpose of intensity-based image registration is to further align the two images based on their intensity distributions. Instead of applying the geometric transformation through feature-based image registration, intensity-based image registration is a non-rigid transformation process that has been widely applied in medical imaging [35] and remote sensing [36]. Here, an example is presented using two images of the same hand under different poses to illustrate the principle. Figure 4a,b are two images (denoted as Image 1 and 2) taken by a smartphone camera (4th generation Moto G Play) with a resolution of 3264 pixels × 2448 pixels. Then, both images were downsized to a lower resolution of 327 pixels × 245 pixels. Due to the different hand poses in these two images, feature-based image registration would face difficulties in aligning the two images. Here, we adopt the algorithm proposed by Thirion [37] to non-rigidly register Image 2 to Image 3 (Figure 4c). A typical three-level pyramid with 500, 400, and 200 iterations is adopted during this procedure. Figure 4d,e further evaluates the registration errors through intensity comparisons. Instead of misalignment of the unregistered images (Image 1 and 2), the two images now are well aligned after the registration (Figure 4e).

Despite the great performance of intensity-based image registration, registration errors may still occur if abrupt intensity changes occur, as shown in Image 2. As an illustration, we intentionally changed the location of the ring on the ring finger during the two image acquisitions, where the ring in Image 2 is closer to the fingertip. Such an action induces abrupt intensity changes in a localized region, leading to registration errors as shown in Figure 4f. However, from the perspective of detecting bolt loosening, such registration errors can be utilized for identifying discrepancies between two images, serving as good features for bolt loosening detection.

### 2.4. Feature Enhancement

**Figure 5:**
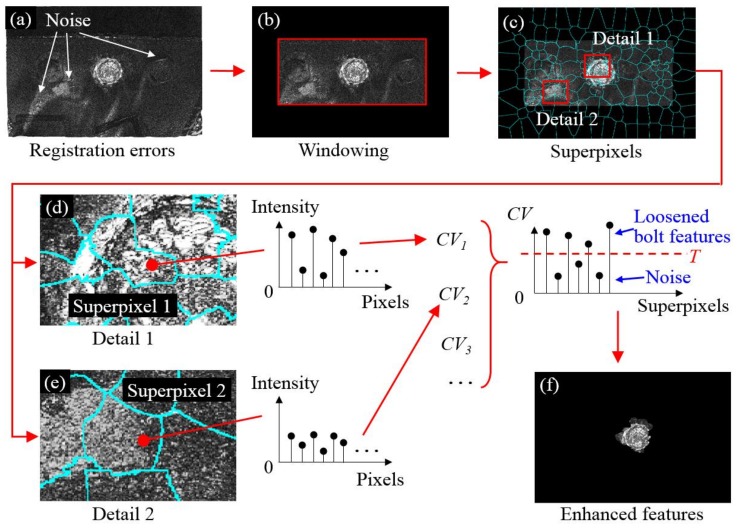
Methodology of feature enhancement: (**a**) registration errors; (**b**) registration errors after windowing; (**c**) segmentation of the registration errors by superpixels; (**d**) detail of Superpixel 1; (**e**) detail of Superpixel 2; and (**f**) registration errors after feature enhancement. The brightness of all figures is enhanced for demonstration purposes.

Once two image registration processes are completed successively, the loosened bolt can be identified through registration errors as shown in Figure 5a. Nevertheless, directly identifying the loosened bolt still requires human intervention as the loosened bolt is surrounded by noise content (Figure 5a). Now, our focus is placed on removing the noise content so that the bolt loosening features around Bolt 2 can be enhanced. A number of image processing techniques have been adopted in this procedure. First, a rectangular window is applied to the registration errors (Figure 5a) so that unrelated results can be filtered out by assigning 0 intensity to the pixels outside the window. The dimensions and location of the window are predefined as the same sizes of the ROI prior to feature-based image registration.

Next, an image segmentation method [38] is performed to segment registration errors (Figure 5b) into a series of localized regions, termed superpixels, as shown in Figure 5c. For each superpixel *i*, the coefficient of variation of intensities at all pixels within this superpixel is computed and is denoted as *CVi*. Then, by applying a cutoff threshold *T*, the noise content can be eliminated from the registration errors so that the bolt loosening features can be preserved (Figure 5f). To explain, suppose two typical superpixels are selected in Figure 5c where Superpixel 1 is from the loosened bolt and Superpixel 2 represents the noise content. As shown in Figure 5d,e, the magnitudes of the intensities around the loosened bolt change dramatically, such as Superpixel 1, while transit smoothly in other regions, such as Superpixel 2. In this regard, extracting the *CVs* of the superpixels can efficiently separate the loosened bolt from its background noise. Hence, a feature enhancement algorithm is proposed by assigning 0 intensity to superpixels whose *CVs* are less than a predefined threshold *T*. For superpixels with *CVs* that are larger than the predefined threshold *T*, no action is required. Utilizing this algorithm, the noise content can be removed and the final result is shown in Figure 5f.

### 2.5. Result Implementation

**Figure 6:**
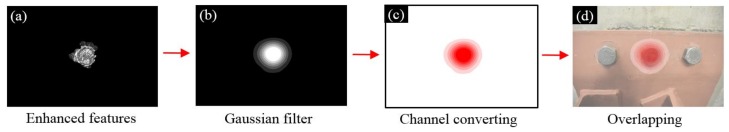
Methodology of the result implementation: (**a**) enhanced features; (**b**) result after applying the Gaussian filter; (**c**) convert the filtered result to RGB channels; and (**d**) overlapping with the original image. The brightness of Figure 6a is enhanced for demonstration purposes.

The purpose of result implementation is to map the bolt loosening features (Figure 6a) to the original input image so that the loosened bolt can be easily visualized. To achieve this goal, a two-dimensional Gaussian filter was applied to Figure 6a to blur the bolt loosening features (Figure 6b). Then, the filtered bolt loosening features are further converted to RGB channels using the following rules: (1) black color in Figure 6b is converted into white color; and (2) white color in Figure 6 is converted into red color. Finally, by setting up the transparency levels and overlapping RGB channels to the original input image, the loosened bolt can be successfully identified (Figure 6d).

## 3. Validations

To validate the proposed approach, three experimental tests were conducted in the laboratory. The digital camera described in Section 2.1 was adopted for image acquisition. The resolution of the collected input images was 6000 pixels × 4000 pixels. Ambient lighting conditions were applied to all the tests during image acquisition. The bolts in the tests were made of ASTM A325 steel with a diameter of 19.05 mm (3/4 in.). These are a common type of high-strength bolts applied in steel constructions in the United States. Shi–Tomasi features and the KLT tracker were adopted for feature-based image registration.

**Figure 7:**
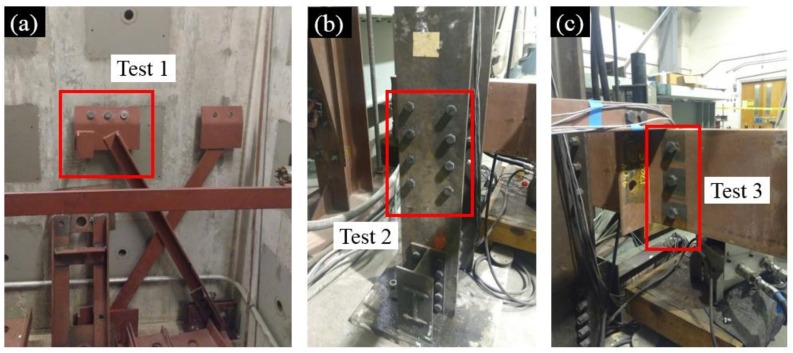
Tested steel joints for three experiments: (**a**) steel joints of Test 1; (**b**) steel joints of Test 2; and (**c**) steel joints of Test 3. The red blocks indicate the monitored bolts.

Figure 7 shows the tested steel joints. The steel joint in Test 1 was from a gusset plate in a cross frame; the steel joint in Test 2 was a steel column flange; and the steel joint in Test 3 was a web region of a steel girder. Table 1 summarizes the different testing parameters in the three experiments in which the total number of bolts, number of loosened bolts, surface textures, and camera orientations vary in order to validate the performance of our approach. The MATLAB Computer Vision System Toolbox [39] was adopted for applying all the algorithms mentioned in Section 2.

**Figure 8:**
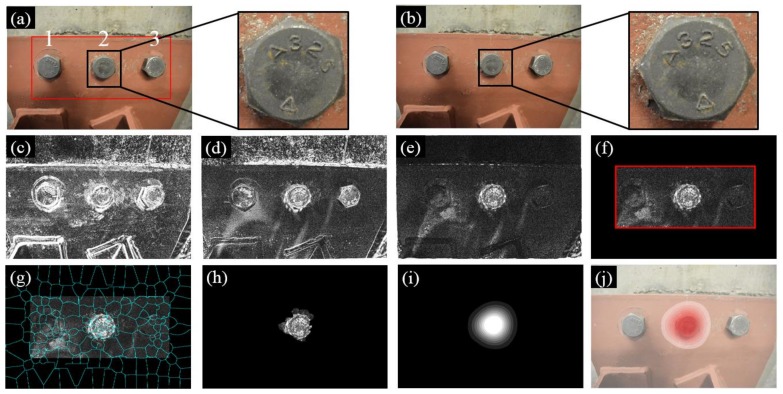
Experimental results of Test 1: (**a**) Image 1 taken at the first inspection period; (**b**) Image 2 taken at the second inspection period; (**c**) initial errors between Image 1 and 2; (**d**) errors after feature-based image registration; (**e**) errors after intensity-based image registration; (**f**) windowing applied to the registration errors; (**g**) registration errors segmented into superpixels; (**h**) feature enhancement; (**i**) Gaussian filtering; and (**j**) result implementation. The brightness of Figure 8c–h is enhanced for demonstration purposes.

Figure 8 summarizes the experimental results of Test 1 where the three bolts in the gusset plate in Image 1 are denoted as Bolt 1, 2, and 3 (Figure 8a). During the inspection interval, Bolt 2 was rotated and then Image 2 was collected as shown in Figure 8b. Figure 8c shows the initial intensity comparison of the two images where significant errors can be found due to the different camera poses. To improve the matching performance, the feature-based and intensity-based image registrations were applied successively and their registration errors are shown in Figure 8d,e. The feature-based image registration is based on the ROI defined near the group of bolts (see the red block in Figure 8a). Then a number of image processing techniques were further applied in order to enhance the bolt loosening features and visualize the loosened bolt as discussed in Section 2.4 and Section 2.5. These techniques included windowing (Figure 8f), superpixel segmentation (Figure 8g), feature enhancement (Figure 8h), Gaussian filtering (Figure 8i), and result overlapping (Figure 8j).

**Figure 9:**
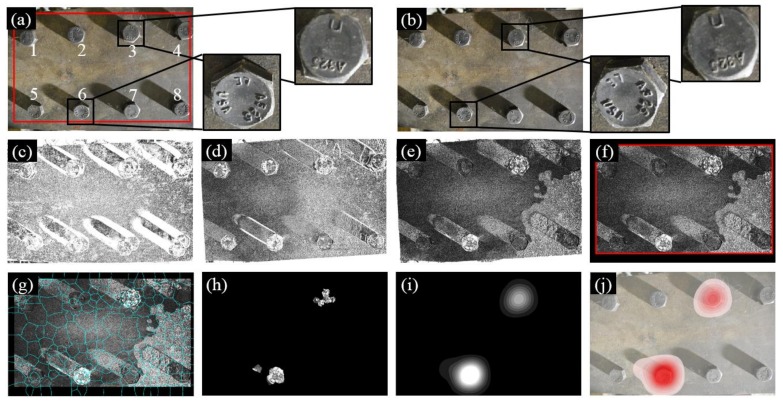
Experimental results of Test 2: (**a**) Image 1 taken at the first inspection period; (**b**) Image 2 taken at the second inspection period; (**c**) initial errors between Image 1 and 2; (**d**) errors after feature-based image registration; (**e**) errors after intensity-based image registration; (**f**) windowing applied to the registration errors; (**g**) registration errors segmented into superpixels; (**h**) feature enhancement; (**i**) Gaussian filtering; and (**j**) result implementation. The brightness of Figure 9c–h is enhanced for demonstration purposes.

Figure 9 illustrates the experimental results of Test 2. Instead of three bolts, a total of eight bolts were adopted in Test 2 and two of them (i.e., Bolts 3 and 6) experienced rotations during the inspection, as shown in Figure 9b. Nevertheless, our approach is still able to identify the loosened bolts as illustrated in Figure 9j.

**Figure 10:**
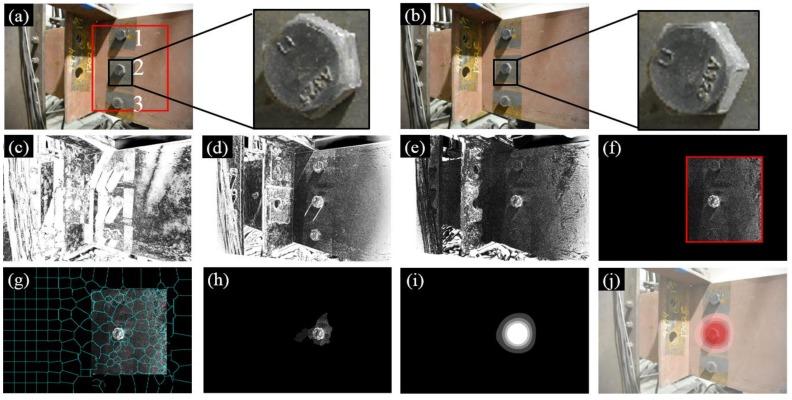
Experimental results of Test 3: (**a**) Image 1 taken at the first inspection period; (**b**) Image 2 taken at the second inspection period; (**c**) initial errors between Image 1 and 2; (**d**) errors after feature-based image registration; (**e**) errors after intensity-based image registration; (**f**) windowing applied to the registration errors; (**g**) registration errors segmented into superpixels; (**h**) feature enhancement; (**i**) Gaussian filtering; and (**j**) result implementation. The brightness of Figure 10c–h is enhanced for demonstration purposes.

In Test 3, multiple conditions were varied in order to validate the performance of our approach. In particular, the orientation of the camera was skewed to the monitored surface instead of parallel as in Tests 1 and 2. The surface treatment of the structural surface, on the other side, was a combination of painted and unpainted, as can be seen in Figure 7c. Prior to processing the images, the ROI (red block in Figure 10a) should be selected to only cover the detected bolts and their adjacent structural surface, while excluding any background that is far from the monitored surface. The benefit of such a selection is twofold: (1) the ROI can facilitate a feature-based image registration process by specifying a localized region for matching potential correspondences; (2) the ROI can also exclude unnecessary registration errors during the feature enhancement procedure (see Figure 10f). As shown in Figure 10j, the loosened bolt (i.e., Bolt 2) can be detected.

As a summary of these experimental results, our approach can successfully detect and localize single or multiple loosened bolts from a group of bolts, regardless of the total number of bolts, structural surface textures, or camera orientation. The success of our approach, however, does rely on tuning the cutoff threshold *T*, a parameter in the feature enhancement algorithm introduced in Section 2.4. As shown in Table 1, *T* was 50 in both Test 1 and Test 3 but increased to 200 in Test 2. This is because more noise content occurred in the registration errors in Test 2, as demonstrated in Figure 9f. A detailed discussion about the effect of *T* will be presented in Section 4.4.

## 4. Robustness Tests

While the performance of our approach has been validated in Section 3 through three experimental tests, robustness is another important concern associated with many approaches in the computer vision-based SHM. In this regard, detailed discussions about various aspects of the robustness of our approach are offered in this section.

### 4.1. Input Image Resolution

**Figure 11:**
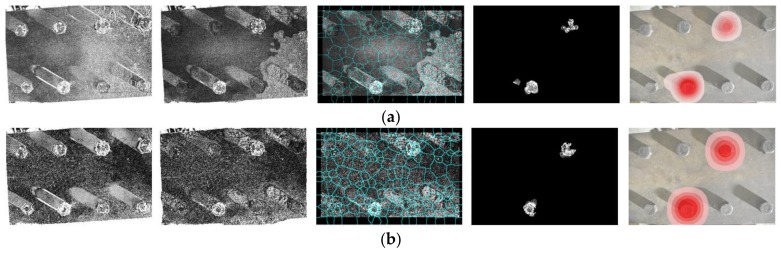
Comparison of bolt loosening detection results with input image resolutions of (**a**) 6000 pixels × 4000 pixels and (**b**) 750 pixels × 500 pixels. Five columns of Figure 11 represent (1) errors of feature-based image registration; (2) errors of intensity-based image registration; (3) superpixel segmentation; (4) feature enhancement; and (5) final result. The brightness of the images in the first to fourth columns is enhanced for demonstration purposes.

The resolution of input images was 6000 pixels × 4000 pixels for the experimental tests in Section 3. However, lower resolution images were able to give successful results. A parametric study was performed by downsizing the original input images and repeating the bolt loosening detection procedure. Two input images of Test 2 in Section 3 were adopted. Two image resolutions were selected including (1) 6000 pixels × 4000 pixels (the original) and (2) 750 pixels × 500 pixels. Figure 11 summarizes the bolt loosening detection results for each scenario.

As shown in the first and second columns in Figure 11, similar registration errors can be found after two image registrations processes. The superpixel segmentation also demonstrates robust performance, despite a slightly different segmentation layout in each scenario. Nevertheless, two loosened bolts were consistently identified regardless of the image resolutions. This further verifies that the key components in our approach are insensitive to input image resolutions. This finding allows reductions in data storage and computational cost through the utilization of lower resolution images.

### 4.2. Rotation Angles of Bolt Head

**Figure 12:**
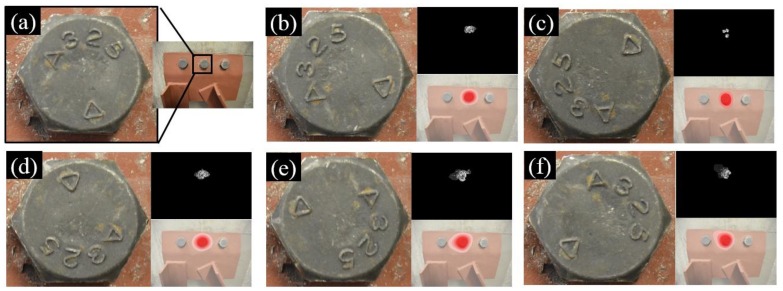
Detection results of a loosened bolt with different rotation angles where (**a**) is the initial status of the loosened bolt; (**b**–**f**) are the detection results when the loosened bolt is subjected to counterclockwise rotation of 60, 120, 180, 240, and 300 degrees, respectively. The three subfigures in Figure 12b–f represent loosened bolt (**right**), enhanced bolt loosening features (**top right**), and detection result (**bottom right**).

The robustness of our approach against different rotation angles of the bolt head is demonstrated in Figure 12. The setup of Test 1 in Section 3 was adopted in this investigation, where the middle bolt in Figure 8a was subjected to a series of counterclockwise rotations of 60, 120, 180, 240, and 300 degrees, respectively. Images were taken at the initial stage with the unloosened bolt (Figure 12a) and the stages thereafter (Figure 12b–f). For these images with the loosened bolts, they were further paired with the initial images for the purpose of bolt loosening detection. All images were collected by the digital camera described in Section 2.1. Image planes are parallel to the monitored surface. To enhance the image processing efficiency, the original input images were downsized to 1500 pixels × 1000 pixels. As can be seen in Figure 12, our approach consistently localized the loosened bolt under different rotation angles.

### 4.3. Features for Tracking

The feature-based image registration adopted in this study also shows great potential to be applied to other research fields in the SHM community, such as targetless displacement monitoring of civil structures [11,40]. One research question associated with the vision-based SHM community is about the types of features that are suitable for targetless tracking. Several discussions are available in the literature [23,41]. Here an investigation of feature type selection is provided in the context of bolt loosening detection in this study.

**Figure 13:**
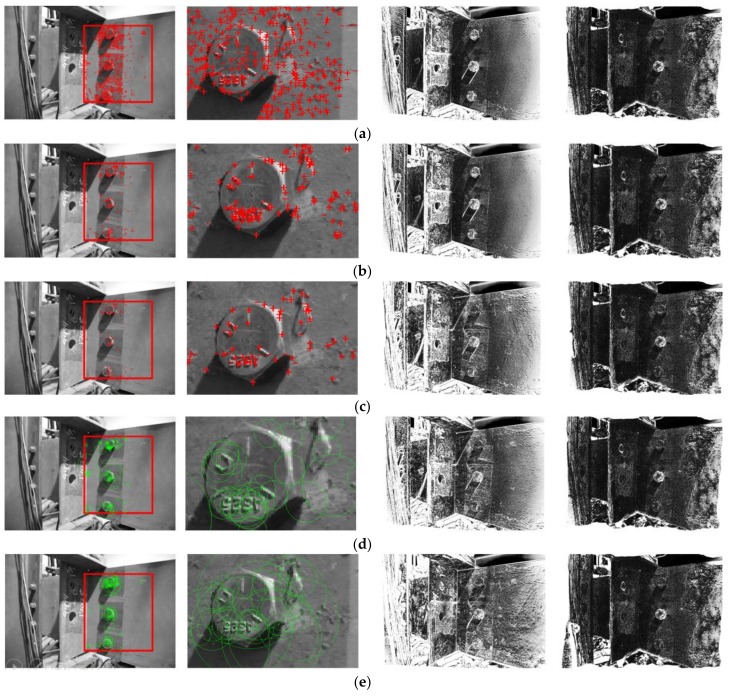
Comparison of bolt loosening detection results with different types of features including (**a**) Shi–Tomasi; (**b**) features from accelerated segment test (FAST); (**c**) Harris–Stephens; (**d**) binary robust invariant scalable keypoints (BRISK); and (**e**) speeded up robust features (SURF). Four columns of Figure 13 represent (1) features in the ROI; (2) detailed look of features around the top bolt; (3) errors after feature-based image registration; and (4) errors after intensity-based image registration. The brightness of the images in the third and fourth columns is enhanced for demonstration purposes.

Here we compare Shi–Tomasi features (adopted in this study) with other common types of features from the literature including features from accelerated segment test (FAST) [42], Harris–Stephens [43], binary robust invariant scalable keypoints (BRISK) [44], and speeded up robust features (SURF) [45]. Briefly, two input images of Test 3 in Section 3 (Figure 10a,b) were adopted in this investigation. Five different types of features were extracted in the first input image within the ROI as shown in the first column of Figure 13. Despite the total number and locations of these features (see the second column in Figure 13), feature-based image registration was successfully performed as shown in the third column of Figure 13. As shown in the last columns of Figure 13, registration errors can be significantly reduced after intensity-based image registration. In this regard, the loosened bolt can be consistently identified by our approach regardless of feature type. This result further validates the robustness of a feature-based image registration process in the proposed approach.

### 4.4. Cutoff Threshold T of Image Segmentation

**Figure 14:**
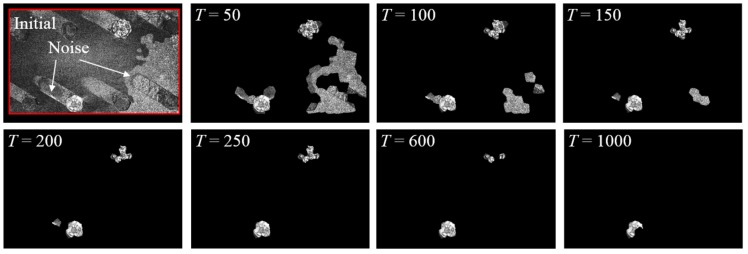
The initial bolt loosening features and results after applying a cutoff threshold *T* with magnitudes of 50, 100, 150, 200, 250, 600, and 1000, respectively. The brightness of all images is enhanced for demonstration purposes. The red block in Figure 14a is the ROI.

As discussed in Section 2.4, a cutoff threshold *T* is introduced to eliminate the noise content from the registration errors. Here, a detailed investigation was performed to demonstrate the sensitivity of *T* in the process of feature enhancement. Two input images in Test 2 of Section 3 were adopted for this investigation and the results are shown in Figure 14. As shown in the figure, a larger cutoff threshold *T* can eliminate noise content in the initial registration errors; however, the bolt loosening features may also be deleted (see the subfigure when *T* = 1000). On the other hand, a smaller cutoff threshold *T* can preserve bolt loosening features. As a tradeoff, noise content may exist as shown in the second subfigure when *T* = 50, leading to challenges in localizing the loosened bolts. For such reasons, *T* = 200 was adopted in the experiment in Section 2. Selecting a region of cutoff threshold *T* from 200 to 600 may also be achievable for this particular dataset. A practical approach for determining the optimal cutoff threshold *T* would be a trial-and-error procedure. An initial *T* = 50 was suggested for the tests in this study and can be further adjusted based on the tuning result.

### 4.5. Lighting Condition

**Figure 15:**
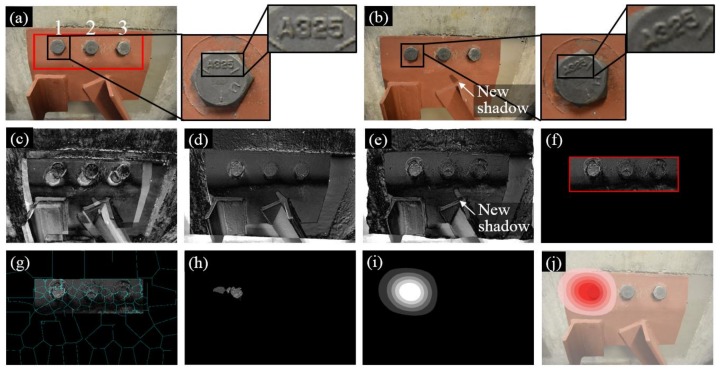
Experimental results under varying lighting conditions: (**a**) Image 1 taken during the first inspection period; (**b**) Image 2 taken during the second inspection period; (**c**) initial errors between Image 1 and 2; (**d**) errors after feature-based image registration; (**e**) errors after intensity-based image registration; (**f**) windowing applies to the registration errors; (**g**) registration errors segmented into superpixels; (**h**) feature enhancement; (**i**) Gaussian filtering; and (**j**) result implementation. The brightness of Figure 10c–h is enhanced for demonstration purposes. The red block in Figure 15a is the ROI.

Lighting condition is another important parameter. Results reported so far were from two input images taken under similar lighting conditions. Here, a further investigation was performed with varying lighting conditions. The setup of Test 1 was adopted in this experiment. As shown in Figure 15b, the lighting condition was varied by adding an additional light source from a floor lamp, leading to slight changes in the shadows in the second input image. Bolt 1 (Figure 15a) experienced a rotation under the inspection interval. Nevertheless, our approach still detected the loosened bolt under such a condition.

Despite the success of our approach in this particular investigation, a significant change in the lighting conditions around the bolts could affect the performance of this approach. The reason is that significant changes in lighting conditions would provoke extensive intensity changes, inducing excessive registration errors. For instance, the new shadow of the angle caused by the lighting change denoted in the second input image (Figure 15b) cannot be eliminated by the two image registration processes and, hence, appears in the registration error in Figure 15e. If such a change in the lighting conditions occurs around the bolts, it would affect the robustness of this approach. Errors caused by changes in lighting conditions are common issues associated with vision-based SHM approaches. Similar challenges have been reported by other researchers [46,47,48].

### 4.6. Nut Loosening

**Figure 16:**
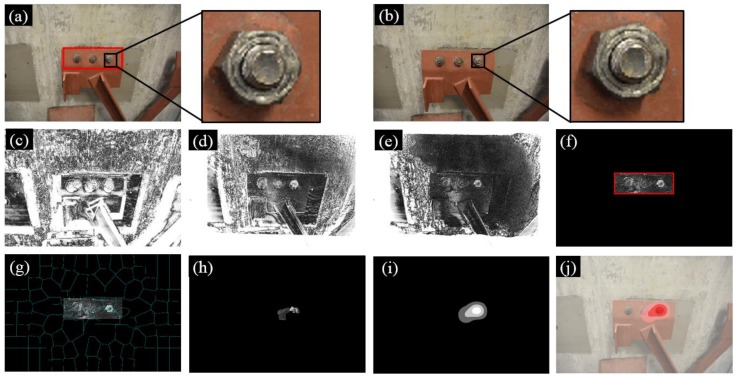
Experimental results under nut loosening: (**a**) Image 1 taken during the first inspection period; (**b**) Image 2 taken during the second inspection period; (**c**) initial errors between Image 1 and 2; (**d**) errors after feature-based image registration; (**e**) errors after intensity-based image registration; (**f**) windowing applied to the registration errors; (**g**) registration errors segmented into superpixels; (**h**) feature enhancement; (**i**) Gaussian filtering; and (**j**) result implementation. The brightness of Figure 10c–h is enhanced for demonstration purposes. The red block in Figure 16a is the ROI.

Nut loosening is another common phenomenon caused by self-loosening of the bolt. Utilizing the proposed methodology, nut loosening can also be detected. Figure 16 illustrates an example through the setup of Test 1 in Section 3. Instead of bolt heads, nuts were installed at the facial side of the gusset plate, as shown in Image 1 (Figure 16a). The third nut from the left experienced a counterclockwise rotation (about 15 degrees) during the inspection interval and then Image 2 was collected (Figure 16b). The result indicates that our approach is able to identify the loosened nut (Figure 16j).

### 4.7. Bolt Type

**Figure 17:**
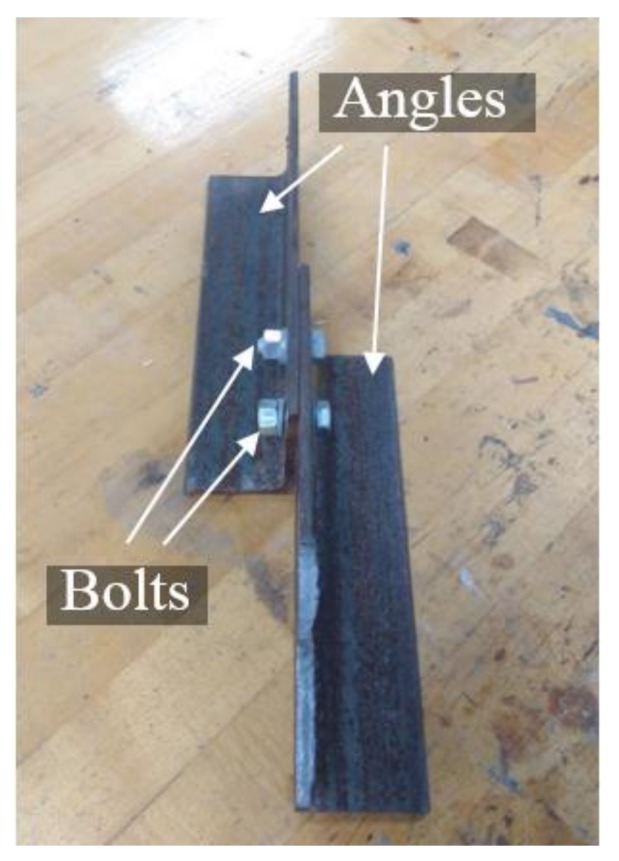
The test specimen placed on a table.

**Figure 18:**
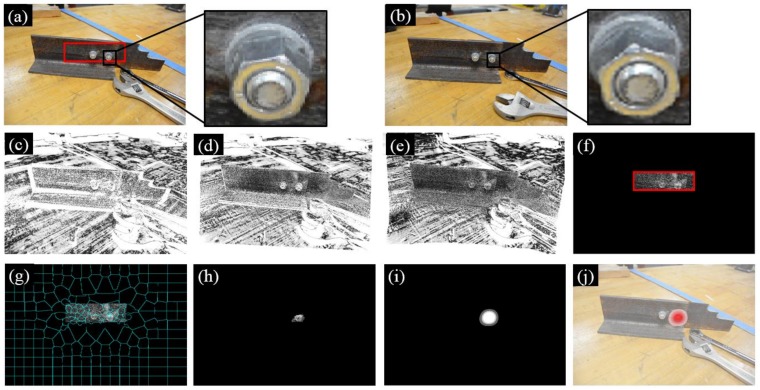
Experimental results under a new bolt type: (**a**) Image 1 taken during the first inspection period; (**b**) Image 2 taken during the second inspection period; (**c**) initial errors between Image 1 and 2; (**d**) errors after feature-based image registration; (**e**) errors after intensity-based image registration; (**f**) windowing applied to the registration errors; (**g**) registration errors segmented into superpixels; (**h**) feature enhancement; (**i**) Gaussian filtering; and (**j**) result implementation. The brightness of Figure 10c–h is enhanced for demonstration purposes. The red block in Figure 18a is the ROI.

A validation of our approach for a different bolt type is demonstrated in Figure 18a. A double angle steel joint with two bolts was adopted in this experiment. The dimensions of the double angles were 2L76.2mm × 50.8 mm × 4.8 mm (2L3in. × 2in. × 3/16in.). The diameter of each bolt was 7.9 mm (5/16 in.), which is much smaller than the bolt (19.05 mm) applied in Section 3. Figure 17 shows the test specimen.

Figure 18 shows the experimental results. During the experiment, the second nut from the left was rotated about 30 degrees in the counterclockwise direction, as shown in Image 2 (Figure 18b). Such a rotation leads to registration errors around the loosened nut, which was detected by our approach, as shown in Figure 18j.

### 4.8. Gap Caused by Nut Loosening

**Figure 19:**
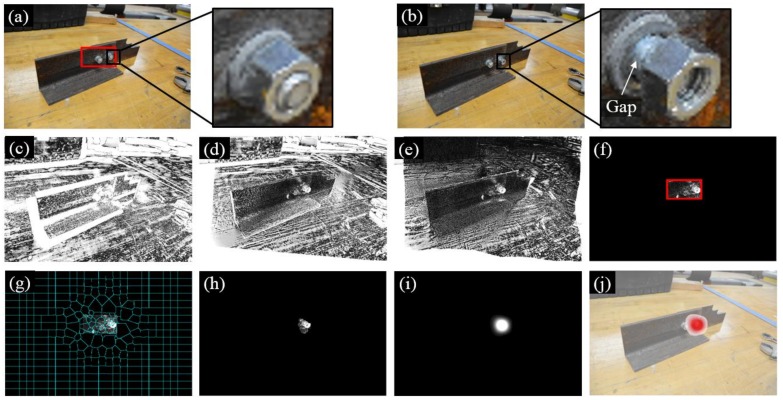
Experimental results under a gap caused by nut loosening: (**a**) Image 1 taken during the first inspection period; (**b**) Image 2 taken during the second inspection period; (**c**) initial errors between Image 1 and 2; (**d**) errors after feature-based image registration; (**e**) errors after intensity-based image registration; (**f**) windowing applied to the registration errors; (**g**) registration errors segmented into superpixels; (**h**) feature enhancement; (**i**) Gaussian filtering; and (**j**) result implementation. The brightness of Figure 10c–h is enhanced for demonstration purposes. The red block in Figure 19a is the ROI.

Instead of finding the rotation of the bolts’ heads and nuts, an alternative strategy for bolt loosening detection is to identify the change in the gap between the nut and the bolted surface. This strategy would be particularly useful for practical implementation if the front view of the bolt heads and/or nuts are difficult to obtain in field conditions (e.g., the space in front of the monitored structure is occupied by other objects). As demonstrated in Figure 19b, the loosened nut results in a gap at the second bolt. The changes in intensities associated with this outward movement of the nut become good features for bolt loosening detection (Figure 19j).

## 5. Discussions

### 5.1. Computational Cost

The computational cost of our approach is related to the resolution of input images. The most time-consuming process in this approach is the intensity-based image registration. For a desktop computer (16 GB RAM, 3.1 GHz CPU), the computational time is 660 s if the input image resolution is 6000 pixels × 4000 pixels, and can be significantly shortened to 20 s in the case of a lower input image resolution (750 pixels × 500 pixels). A detailed summary of the computational cost under the same desktop computer against different image resolutions is shown in Table 2. Based on the discussion in Section 4.1, we recommend applying lower resolution input images to reduce the computational cost.

### 5.2. Limitations

Despite the success of detecting loosening bolts presented in this study, several limitations still exist in our approach. The main limitation is that the lighting conditions and camera poses need to be similar during the two inspection periods. Significant changes of camera poses (e.g., the first input image is taken from the front view of the monitored structure while the second input image is taken from the side view) would cause failure of the feature-based image registrations, leading to extensive misalignments (i.e., registration errors). Adjusting such misalignments is beyond the capabilities of our approach. Nevertheless, small differences of the camera pose are generally acceptable. A successful example can be found in Section 4.6 in which the camera was closer to the structure in the first input image. The sensitivity to lighting conditions of our approach has been discussed in Section 4.5. Despite the robustness of our approach as demonstrated in this example, drastically changing the lighting condition around the bolts between two inspections (e.g., the first input image is taken under ambient lighting conditions while the second input image is collected with a flashlight) would affect the performance of our approach. Similar challenges with lighting conditions have been reported in many studies on vision-based SHM [46,47,48].

Due to the nature of the intensity-based image registration applied in this study, any small misalignment between two input images will be forced to match each other. In this regard, our approach is not able to detect the loosened bolt subjected to very small rotations of bolt heads and/or nuts, as misalignments caused by small rotations will be eliminated by the intensity-based image registration. For this particular study, our approach would fail to detect bolt loosening if the rotation of the bolt head and/or nut is less than 10 degrees. A similar limitation was also reported in another computer vision-based bolt loosening detection method [26]. Finally, our approach provides binary detection results and does not quantify the rotation angles of the bolt heads and/or nuts.

## 6. Conclusions

In this study, we have proposed a computer vision-based bolt loosening detection method through image registrations. Our approach starts with the collection of two input images of the detected steel joint during different inspection periods, followed by identification of the loosened bolt through comparison, enhancement, and visualization of differential features caused by bolt loosening. Specifically, we first adopted feature-based and intensity-based image registration processes to eliminate misalignments around a group of bolts between the two input images. Then, we established a feature enhancement method to remove noise content in the registration errors. Finally, a damage visualization approach was applied to localize the bolt loosening features in the original input image.

To validate the performance of our approach, three experimental tests were performed in the laboratory by utilizing a gusset plate on a cross frame, a column flange, and a girder web. The test results have verified that our approach can detect single or multiple loosened bolts from a group of bolts, regardless of the total number of bolts, structural surface textures, and camera orientations. Furthermore, the robustness of our approach has been investigated in the contexts of input image resolutions, rotation angles of the bolt head, cutoff threshold *T*, lighting conditions, nut loosening, different bolt types, and the gap caused by nut loosening. In addition, registration performances under different types of features have been compared to demonstrate the flexibility of our approach.

Finally, computational cost and sensitivities of the proposed approach against lighting conditions, camera poses, and bolt rotation angles have been discussed. The results of the proposed method are presented in a way for easy interpretation, such that direct actionable decisions can be made for conducting condition-based maintenance procedures, such as tightening or replacing the loosened bolts, to ensure the structural integrity. Our future work will focus on enhancing the robustness of our approach against lighting conditions and camera poses through quantitative investigations and combing our approach with the UAV platform for achieving autonomous field inspections of civil structures.

## Figures and Tables

**Table 1:** Test matrix.

Test Number	Description	Total Bolts	Loosened Bolts	Structural Surface	Cutoff Threshold *T*	Relation of Image Plane to the Monitored Surface
Test 1	Gusset plate	3	1 (Bolt 2 in Figure 8a)	Painted	50	Parallel
Test 2	Column flange	8	2 (Bolt 3 and 6 in Figure 9a)	Unpainted	200	Parallel
Test 3	Girder web	3	1 (Bolt 2 in Figure 10a)	Mixed	50	Skewed

**Table 2:** Computational cost.

Image Resolution	Duration of Computation
6000 pixels × 4000 pixels	660 s
3000 pixels × 2000 pixels	182 s
1500 pixels × 1000 pixels	65 s
750 pixels × 500 pixels	20 s
